# Genetic relatedness and molecular characterization of multidrug resistant *Acinetobacter baumannii *isolated in central Ohio, USA

**DOI:** 10.1186/1476-0711-8-21

**Published:** 2009-06-17

**Authors:** Vijaya B Srinivasan, Govindan Rajamohan, Preeti Pancholi, Kurt Stevenson, Daniel Tadesse, Prapas Patchanee, Mario Marcon, Wondwossen A Gebreyes

**Affiliations:** 1 Department of Veterinary Preventive Medicine, College of Veterinary Medicine, Columbus, Ohio, USA; 2 Institute of Microbial Technology, CSIR, Sector 39A, Chandigarh, India; 3 Department of Pathology, College of Medicine, The Ohio State University, Columbus, Ohio, USA; 4 Department of Internal medicine, Division of Infectious Diseases, College of Medicine, The Ohio State University, Columbus, Ohio, USA; 5 Department of Clinical Epidemiology, The Ohio State University Medical Center, Columbus, Ohio, USA; 6 Department of Laboratory Medicine, Nationwide Children's Hospital, Columbus, Ohio, USA

## Abstract

**Background:**

Over the last decade, nosocomial infections due to *Acinetobacter baumannii *have been described with an increasing trend towards multidrug resistance, mostly in intensive care units. The aim of the present study was to determine the clonal relatedness of clinical isolates and to elucidate the genetic basis of imipenem resistance.

**Methods:**

*A. baumannii *isolates (n = 83) originated from two hospital settings in central Ohio were used in this study. Pulsed-field gel electrophoresis genotyping and antimicrobial susceptibility testing for clinically relevant antimicrobials were performed. Resistance determinants were characterized by using different phenotypic (accumulation assay for efflux) and genotypic (PCR, DNA sequencing, plasmid analysis and electroporation) approaches.

**Results:**

The isolates were predominantly multidrug resistant (>79.5%) and comprised of thirteen unique pulsotypes, with genotype VII circulating in both hospitals. The presence of *bla*_OXA-23 _in 13% (11/83) and IS_*Aba1 *_linked *bla*_OXA-66 _in 79.5% (66/83) of clinical isolates was associated with high level imipenem resistance. In this set of OXA producing isolates, multidrug resistance was bestowed by *bla*_ADC-25_, class 1 integron-borne aminoglycoside modifying enzymes, presence of sense mutations in *gyrA*/*parC *and involvement of active efflux (with evidence for the presence of *adeB *efflux gene).

**Conclusion:**

This study underscores the major role of carbapenem-hydrolyzing class D β-lactamases, and in particular the acquired OXA-23, in the dissemination of imipenem-resistant *A. baumannii*. The co-occurrence of additional resistance determinant could also be a significant threat.

## Background

*Acinetobacter baumannii *is a rapidly emerging nosocomial pathogen and causes severe infections that include bacteremia, pneumonia, meningitis, urinary tract and wound infections [1]. It has now become a major cause of hospital-acquired infections worldwide due to its remarkable propensity to rapidly acquire resistance determinants to a wide range of antibacterial agents [2]. Of note, increasing resistance to carbapenems has been observed worldwide in the past decade [3]. Carbapenemase production is the most described mechanism of resistance to carbapenems [4]. The carbapenemases in *A. baumannii *have belonged to the *bla*_OXA-23-_, *bla*_OXA-24-_, and *bla*_OXA-58- _type class D family of serine β-lactamases and IMP/VIM class B metallo-β-lactamases [3,4]. The upstream of OXA type class D carbapenemases in *Acinetobacter *is often associated with insertion sequence (IS), IS*Aba1 *and other IS may modulate the expression and transfer of OXA-type carbapenemase genes [5-10]. IS are mobile genetic elements known to affect the evolutionary pattern of bacterial genomes. Upon integration, IS elements may cause DNA insertions/deletions, chromosomal rearrangement, modulate the expression of neighbouring genes and, thereby, influence the phenotype of a bacterium [11].

Numerous outbreaks caused by multidrug-resistant (MDR) *A. baumannii *from different parts of United States are appearing very rapidly [12-16]. One of the most poignant instances is the widespread prevalence of MDR *A. baumannii *among personnel returning from military operations in Iraq and Afghanistan [17]. The Infectious Diseases Society of America (IDSA) identified *A. baumannii *among the top seven pathogens threatening our healthcare-delivery system and as a crucial example of unmet medical need [18].

Our phenotypic analysis clearly demonstrated that *A. baumannii *isolates obtained from different hospitals in central Ohio were resistant to all clinically significant antibiotics, including carbapenems (imipenem). The aim of the present study was to determine the clonal relatedness among clinical isolates and the genetic basis for imipenem resistance. Molecular determinants enabling the imipenem resistant strains to exhibit co-resistance to aminoglycosides and fluoroquinolones from this geographical region were delineated.

## Methods

### Study population

*A. baumannii *isolates (n = 83) that originated from two sources were investigated. They consisted of isolates from The Ohio State University Medical Center (referred as MC) (n = 47) and other central Ohio hospitals retrieved from the Ohio Department of Health (referred as ODH) (n = 36) collected during 2005–2007 time period. These isolates were obtained from different Intensive Care Units (ICU) and non-ICUs in the hospitals. The selection criteria of these strains were based on the heterogeneity in their properties such as, geographic origin, time of isolation, levels of resistance to carbapenems, aminoglycosides and fluoroquinolones, thus excluding multiple isolates of the same strain from one locality. Forty-seven isolates of MC were originally isolated from aspirated sputum (24%), BAL (17%), bronchial wash (16%) and other systems including blood (26%), wound (2%) and urinary infections (15%). Thirty-six isolates from ODH were obtained from bronchial wash (37%), sputum (33%), blood (8%), BAL (12%) and remaining 10% from urine and wound. The isolates were obtained from patients belonging to different age groups: 60–90 years (n = 54), 20–50 years (n = 28) and one isolate from a 15-year-old. No additional individual patient data was retrieved as it was beyond the scope of this investigation. Institutional Review Board exemption was obtained prior to retrieval of the isolates from the pathogen bank.

### Bacterial isolation and identification

The 83 *A. baumannii *clinical isolates were identified by using the Vitek 2^® ^automated instrument ID system (BioMérieux, Marcy l'Etoile, France), API 20NE system (BioMerieux, Inc) and NUC 45 Identification Panel (MicroScan^R^, Siemen's Healthcare, Sacramento, CA, USA) and sequencing of the *gyrA *house keeping gene, as described previously [19].

### Minimum Inhibitory Concentration (MIC)

Susceptibilities of *A*.*baumannii *isolates to imipenem, ceftazidime, amikacin, streptomycin, gentamicin, kanamycin, tetracycline, ciprofloxacin and nalidixic acid were tested using broth dilution technique. Multidrug resistance was defined in this analysis as resistance to three or more representatives of the following classes of antibiotics: quinolones (ciprofloxacin and nalidixic acid), extended-spectrum cephalosporins (ceftazidime), aminoglycosides (amikacin, streptomycin, gentamicin, kanamycin), and carbapenems (imipenem). Interpretation was done as per the criteria approved by the Clinical and Laboratory Standards Institute CLSI [20]. *E. coli *ATCC 25922 was used as a reference strain (control) as recommended.

### Pulsed-field gel electrophoresis (PFGE) genotyping

PFGE was performed according to the Centers for Disease Control and Prevention Pulse Net protocol [21] with minor modifications. Fingerprint images were analyzed by Bionumerics software V. 4.61 (Applied Maths NV, Belgium) using dice similarity index for cluster analysis and the unweighted pair group average (UPGMA) for tree building. All isolates with PFGE banding patterns with a similarity index >75% were grouped within the same cluster. Banding patterns were compared with 3.0% optimization and 2.5% band position tolerance.

### PCR amplifications and sequence analyses

Genomic DNA was extracted using DNeasy Tissue kit (Qiagen; Valencia, CA, USA). PCR for evaluating the presence of 13 different β-lactamases, IS_*Aba1*_, IS_1133_, class 1 integrons and its variable region, *int2*, *int3, aphA6*, *qnrA*_1–6_, *qnrB*_1–6 _and *qnrS*_1–2_[22], *tet(A), tet(B) *[23], quinolone resistance determining region (QRDR) of *gyrA*, *parC*, *adeB*, *adeR *and *adeS *genes were carried out using specific primers (Table 1). All amplicons were sequenced bidirectionally using CEQ 8000 (Beckman Coulter Instruments Inc., Palo Alto, CA) capillary electrophoresis system and analyzed by BLAST at . To determine the location and orientation of the insertion sequence (IS) element, combination primers specific to both IS_*Aba1 *_and *bla*_OXA-66 _or *bla*_ADC-25 _were used [24,25].

**Table 1:** Primers used for PCR amplification

	**Primer sequence (5' to 3')**			**Reference**
**Target gene(s)**	**Forward**	**Reverse**	**Size of amplicon**	

bla_TEM-1_	GCACGAGTGGGTTACATCGA	GGTCCTCCGATCGTTGTCAG	310	14
bla_PER-1_	ATGAATGTCATTATAAAAG	TTGGGCTTAGGGCAG	927	14
bla_IMP alleles1-21_	GTTTATGTTCATACWTCG	GGTTTAAYAAAACAACCAC	432	14
bla_VIM alleles_	TTTGGTCGCATATCGCAACG	CCATTCAGCCAGATCGGCAT	500	14
bla_GIM_	ATATTACTTGTAGCGTTGCCAGC	TTAATCAGCCGACGCTTCAG	729	14
bla_SHV_	ATGCGTTATATTCGCCTGTG	TGCTTTGTTATTCGGGCCAA	753	14
bla_SIM_	ATGAGAACTTTATTGATTTT	TTAATTAATGAGCGGCGGTT	741	This study
bla_CTX-M_	ATGATGACTCAGAGCATTCGCCGCT	TCAGAAACCGTGGGTTACGATTTTCG	876	14
bla_ADC_	ATGCGATTTAAAAAAATTTCTTGT	TGGAATACGTTTATTGGTTAACATGA	1081	14
OXA-23-like	TCTGGTTGTACGGTTCAGC	AGTCTTTCCAAAAATTTTG	606	14
OXA-24-like	ATGAAAAAATTTATACTTCC	TTAAATGATTCCAAGATTTTC	828	14
OXA-51-like	ACAGAARTATTTAAGTGGG	GGTCTACAKCCMWTCCCCA	880	14
bla_OXA-58_	ATGAAATTATTAAAAATATTGAGTTTAG	TTATAAATAATGAAAAACACCCAAC	843	14
IS_Aba1_	ATGCAGCGCTTCTTTGCAGG	AATGATTGGTGACAATGAAG	393	14
IS_1133_	ATGACACATCTCAATGAGTTATAT	TTAACACGAATGCAGAAGTTGATG	543	14
ISAb-F1/OXA/IS-R	AGTTGCACTTGGTCGAATGAA	CCATAGCTTTGTTGAGTTTGG	540	22
ISAb-F2/OXA/IS-R	TTGAAAATACGCGCTTGACAGA	CCATAGCTTTGTTGAGTTTGG	1104	22
ISAb-F3/OXA/IS-R	CTCTGTACACGACAAATTTCAC	CCATAGCTTTGTTGAGTTTGG	1384	22
IS_Aba1-_ampC-UP	GACCTGCAAAGAAGCGCTGCATA	TTGGTTCTTTTAAACCATATACC	1502	This study
intI1 *5'CS*	GACGATGCGTGGAGACC	CTTGCTGCTTGGATGCC	300	25
intI1 *3'CS*	ATCGCAATAGTTGGCGAAGT	GCAAGGCGGAAACCCGCC	800	25
Variable region	GGCATCCAAGCAGCAAGC	AAGCAGACTTGACCTGAT	Variable	25
intI2	ACGATGCCTGCTTTTTGTACGGCTGC	CCGTCTATCCTGCTTGCACGATGCA	962	This study
intI3	TCAGCCGGGCGACAAGTGCAAGGCCA	ATGAACAGGTATAACAGAAAT	1041	This study
aphA6	ATGGAATTGCCCAATATTATTC	TCAATTCAATTCATCAAGTTTTA	780	14
tet A	GCGCGATCTGGTTCACTCG	AGTCGACAGYRGCGCCGGC	164	36
tet B	TACGTGAATTTATTGCTTCGG	ATACAGCATCCAAAGCGCAC	206	36
qnrA1 to qnrA6	AGAGGATTTCTCACGCCAGG	TGCCAGGCACAGATCTTGAC	661	37
qnrB1 to qnrB6	GGMATHGAAATTCGCCACTG	TTTGCYGYYCGCCAGTCGAA	562	37
qnrS1 to qnrS2	GCAAGTTCATTGAACAGGGT	TCTAAACCGTCGAGTTCGGCG	605	37
gyrA (QRDR)	AAATCTGCCCGTGTCGTTGGT	GCCATACCTACGGCGATACC	285	14
parC (QRDR)	AAAAATCAGCGCGTACAGTG	CGAGAGTTTGGCTTCGGTAT	276	14
adeB	GGATTATGGCGACAGAAGGA	AATACTGCCGCCAATACCAG	981	This study
adeR	ATGTTTGATCATTCTTTTTCTTTTG	TTAATTAACATTTGAAATATG	687	14
adeS	TTCAACAAGAAGATTGGACC	CTTGCTCAATACGACGG	114	28

^a ^Y = C or T; M = A or C; R = A or G; W = A or T; K = G or T; H = A or C or T.

^b ^QRDR, quinolone resistance-determining region.

### Plasmid analysis

Plasmid DNA was isolated using alkaline lysis method as described before [26]. *Escherichia coli *JM109 electrocompetent cells were transformed with 30 ng of plasmid preparations and were screened on LB plates (Difco, Becton-Dickinson, Sparks, MD) containing different antibiotics, (Sigma, St. Louis, MO): amikacin, streptomycin, gentamicin, kanamycin (10 μg/ml respectively), imipenem (1 μg/ml), ciprofloxacin (1 μg/ml), nalidixic acid (30 μg/ml) and tetracycline (10 μg/ml). Transformants were restreaked on identical plates for confirmation.

### *In vitro *studies to elucidate the occurrence of active efflux

The accumulation of ciprofloxacin was examined as described previously [27]. The efflux pump inhibitors used in this study were carbonyl cyanide *m*-chlorophenylhydrazone (CCCP), verapamil and reserpine, (Sigma, St. Louis, MO); to a final concentration of 25 μg/ml. The growth inhibition assay was done as described previously with minor modifications [28]. *A. baumannii *cultures at mid log phase (OD 600_nm _= 0.2) were inoculated into LB broth containing antimicrobials at different concentrations either alone or with inhibitor. The extent of growth inhibition was analyzed by measuring the absorbance at 600 nm (OD 600_nm_) after 6 hrs of incubation at 37°C. All data from the *in vitro *kinetics and flourimetric assay are presented as means ± the standard error of the mean and calculation of the standard deviation was performed in Excel (Microsoft, USA). The statistical significance was determined using the paired Student's *t-*test. P values of < 0.05 were considered significant.

### Accession numbers

The sequences of *aadB*, *aadB*-*aadA2*, *aacC1*-orfX-orfX'-*aadA1*, *aacA4*-*catB8- aadA2*, *bla*_OXA-66_, *bla*_TEM-1_, *bla*_ADC-25_, *aphA6*, IS_*Aba1*_, *bla*_OXA-23_, QRDR of *gyrA *and *parC *genes obtained in this study were deposited in the GenBank database under the following accession numbers: EU977565, EU977566, EU977567, EU977568, EU977569, EU977570, EU977571, EU977572, EU977573, EU977574, EU977575 and EU977576 respectively.

## Results

### Antimicrobial susceptibility and genotypic diversity

In this study, about 79.5% (66/83) were multi-drug resistant (MDR). Among these, 62 were resistant to ceftazidime and 66 were resistant to imipenem. The imipenem resistant isolates (66/83) were also resistant to kanamycin, amikacin, gentamicin, streptomycin, tetracycline, ciprofloxacin and nalidixic acid. Overall, 7% (6/83) were found resistant only to chloramphenicol and remaining 14.5% (12/83) isolates were pan-susceptible (See Additional file 1).

**Figure 1:**
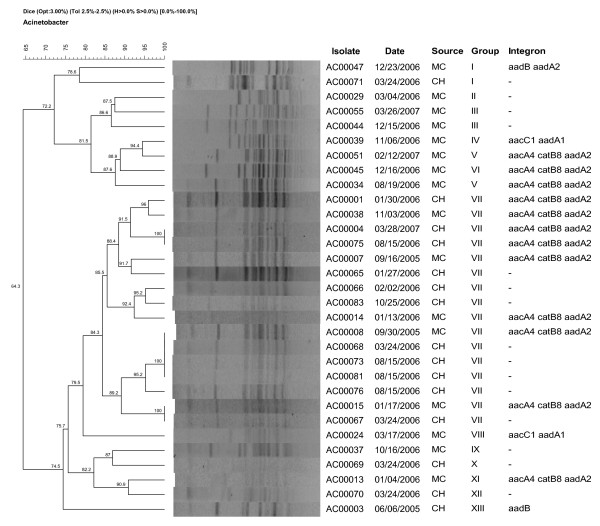
**PFGE profiles of selected strains**. *A. baumannii *isolates representing various resistance profiles from different hospitals and different units were genotyped. Figure is the representation of PFGE fingerprints of thirty one selected isolates. Thirteen genotypes (Group I to XIII) were identified in this study. The percentage of similarities was determined by the Dice's coefficient and UPGMA clustering. Major clusters were formed at the 75% similarity level. * Source abbreviations are MC (Ohio State University Medical Center) and CH (All central Ohio isolates derived from the State Public Health laboratory). The various ICUs in MC include Ross Heart hospital, James Cancer Hospital, Rhodes and Doans hospital and the Emergency Department.

To determine the extent of genotypic diversity among the MDR *A. baumannii*, PFGE was conducted and the isolates were clustered into thirteen major genotypes (I to XIII) at 75% genotypic similarity threshold. Profiles of randomly selected isolates (n = 31) are depicted in Figure 1. Though their genotypes were diverse, majority of the isolates exhibited increased resistance to β-lactams, aminoglycosides and quinolones. Genotype VII was found as the most common cluster type (28/83) that was found circulating in hospitals regardless of origin: 16 isolates from MC (19%) and 12 from ODH (14%).

### Molecular determinants for β-lactam resistance

Eleven imipenem resistant isolates (13%) contained the acquired carbapenem hydrolyzing class D β-lactamase (CHDLs) gene *bla*_OXA-23 _[GenBank: EU977574] (See Additional file 1). The other CHDLs including *bla*_OXA-24 _and *bla*_OXA-58 _like genes could not be identified in this study. Sixty-six imipenem resistant isolates (79.5%) carried IS_*Aba1 *_genetic element preceding the naturally occurring carbapenemase *bla*_OXA-66_, a *bla*_OXA-51 _like gene [GenBank: EU977573, EU977569] (See Additional file 1). However IS_1133 _was not found in this collection.

The cephalosporinase *bla*_ADC-25 _was detected in 62 ceftazidime resistant isolates and PCR mapping indicated that IS_*Aba1 *_was not present upstream to the cephalosporinase identified in this study [GenBank: EU977571] (See Additional file 1).

The class A β-lactamase *bla*_TEM-1 _was found in 37% of the clinical isolates [GenBank: EU977570] (See Additional file 1). The other reported metallo-β-lactamases such as *bla*_SIM_, *bla*_IMP_, *bla*_VIM_, *bla*_GIM _and other β-lactamases including *bla*_SHV_, *bla*_CTX-M_, *bla*_PER_, were not detected in any of our isolates.

### Molecular determinants for aminoglycoside resistance

Class 1 integrons were found in 40% (33/83) of the isolates. The length of the amplicons ranged between 0.75 to 2.5-kb. The 0.75-kb amplicon found in a single isolate carried an aminoglycoside modifying enzyme (AME) *aadB *[Genbank: EU977565]. The 1.6-kb amplicon detected in three isolates with Type I PFGE profile, AC0047, AC0050 and AC0053 harboured *aadB *and *aadA2 *gene cassettes [GenBank: EU977566].

The 2.3-kb amplicon found in 26 isolates carried *aacA4-catB8-aadA2 *gene cassettes [GenBank: EU977568]. In this study, 33% (28/84) of the isolates belonged to PFGE type VII. Of these, 24% (20/84) harboured Class 1 integron with *aacA4*-*catB8-aadA1 *variable region whereas the remaining 9% (8/84) isolates did not harbour integron.

**Figure 2:**
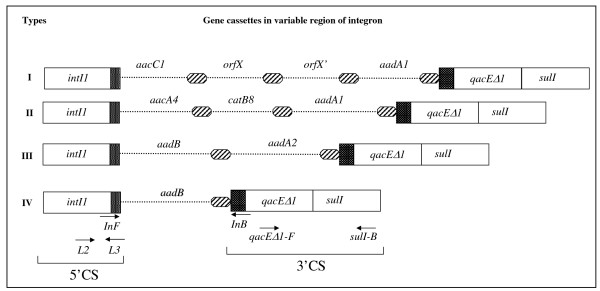
**Schematic representation of different types of gene cassettes identified in the Class 1 integrons in *A. baumannii *strains**. Dotted lines represent the gene cassettes, oval circles the 59-base elements. The *att*I recombination site is shown by the hatched box. Locations of the 5'CS and 3'CS of class 1 integrons and those of the primer pairs, *qacEΔ1-F, Sul1B *and *in-F *(5'CS), *in-B *(3'CS), are shown in the bottom panel. Different types of variable region were found in our collection of isolates: Type I: *accC1*-orfX-orfX'-*aadA1*; n = 3, Type II: *aacA4*-*catB8-aadA1*, n = 26, Type III: *aadB-aadA2*; n = 3 and Type IV: *aadB*; n = 1. *accC1 *(3-N-aminoglycoside acetyltransferase) and *aadB *(2'-O-adenylyltransferase) confers gentamicin resistance, *aadA1 *and *aadA2 *(adenyltransferase) confers resistance to spectinomycin and streptomycin, *aacA4 *(6'-N-acetyltransferase) confers resistance to amikacin, netilmicin and tobramycin, *catB8 *(chloramphenicol acetyltransferase) confers resistance to chloramphenicol. The diagram was not drawn to scale.

A 2.5-kb amplicon obtained in three clinical isolates, AC0023, AC0024 and AC0039 carried *aacC1*-orfX-orfX'-*aadA1 *gene cassettes [GenBank: EU977567] (Figure 2).

The integrases *intI2 *and *intI3 *were not found in any of the isolates. The aminoglycoside resistance gene *aphA6 *was found in 18% of the isolates (15/83) that exhibited different MDR profiles [GenBank: EU977572] (See Additional file 1).

### Plasmid carriage and resistance conferred by plasmids

Plasmids were found in 66 clinical isolates. Plasmids from strains belonging to 13 different pulsotypes were transformed into *E. coli *JM109 and colonies were obtained only on tetracycline containing LB plate. Though the size of transformed plasmids (originally obtained from *A. baumannii *isolates exhibiting various pulsotypes) in the obtained colonies were different (which was 5–9 kb), it was interesting to note that they all harboured *tet*(B) efflux gene (See Additional file 1).

Plasmid borne quinolone resistance *qnr *genes (*qnrA*_1–6_, *qnrB*_1–6 _and *qnrS*_1–2_) could not be identified in this study.

### Characterization of quinolone resistance

In this study, 79.5% of the nalidixic acid and ciprofloxacin resistant isolates (66/83) (See Additional file 1) harboured sense mutations (Serine to Leucine) at the 83^rd ^and 80^th ^positions in *gyrA *and *parC *QRDR respectively [GenBank: EU977575 and EU977576] (See Additional file 1). Additional sense mutations could not be detected elsewhere in these target genes (data not shown).

**Figure 3:**
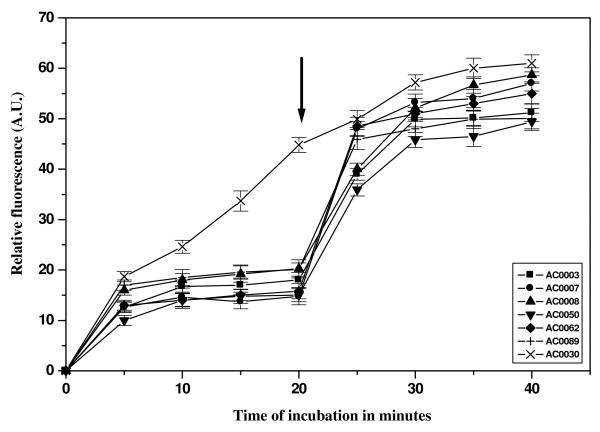
**Accumulation studies with ciprofloxacin**. The fluorescence of the supernatant was measured with spectroflourimeter (LS 55 Fluorescence Spectrometer, 120 V, Perkin-Elmer model) at an excitation 275 nm and emission 440 nm for ciprofloxacin. The results for six representative MDR strains, namely, AC0003, AC0007, AC0008, AC0050, AC0062, AC0089 and one sensitive strain AC0030 are shown here. The graphs reflect the difference in fluorescence displayed by the bacterial cell in the presence and absence of efflux pump inhibitor CCCP. The arrow indicates the time of addition of CCCP. All experiments were carried out at least three times.

**Figure 4:**
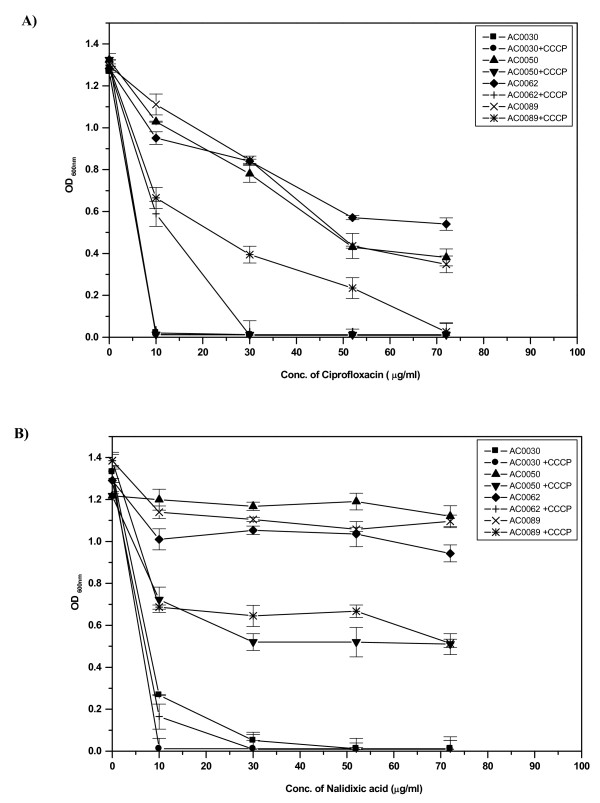
***in vitro *analysis of growth kinetics**. The ability of different MDR clinical isolates namely AC0050, AC0062, AC0089 and sensitive strain AC0030, to grow in the presence of different concentrations of ciprofloxacin (A), nalidixic acid (B), either alone or in the presence of 25 μg/ml of CCCP was monitored in LB broth using a spectrophotometer. Range of results obtained for duplicate experiments are shown by error bars.

To elucidate the role of active efflux [27] ciprofloxacin accumulation studies were performed, and analysis indicated that accumulation of ciprofloxacin at steady state was higher in sensitive strains, which was 2.0 to 2.5 times greater than the amount in MDR strains. Addition of CCCP (25 μg/ml) resulted in the restoration of the fluorescence intensity in MDR strains, eventually increasing its level comparable to that of the sensitive strain (Figure 3). Growth inhibition assay also supported the findings of the CCCP mediated inhibition of ciprofloxacin and nalidixic acid efflux in MDR isolates (Figure 4). Addition of CCCP drastically reduced the MIC for ciprofloxacin in 66 MDR isolates clearly indicating the role of efflux mechanism in conferring quinolone resistance (See Additional file 1).

Specific PCR assays demonstrated that 53% (44/83) of the isolates had the transporter gene *adeB *[29] (See Additional file 1); response regulator *adeR and *its cognate kinase *adeS*. Though 66 strains were quinolone resistant, the *adeB *efflux gene was found in 44 isolates only, this observation indicates that other efflux systems could be involved in mediating quinolone resistance. Single point mutations in *adeR *(Pro116→Leu) and *adeS *(Thr153→Met) known to cause AdeABC constitutive overexpression [30] were not identified in this study.

## Discussion

The study presented here describes the first hospital based outbreak of imipenem resistant *A. baumannii *isolates producing the carbapenem hydrolyzing oxacillinase *bla*_OXA-23 _in central Ohio. OXA-23 producers have been identified as sources of nosocomial outbreaks worldwide, including the United States [1,12,13,16]. Acquired *bla*_OXA-23 _gene is known to be located in peculiar transposon structures, namely, Tn*2006 *(IS*Aba1 *linked) and Tn*2007 *(IS*Aba4 *linked) [8,9,11,31]. Of note, the origin of *bla*_OXA-23 _was recently identified as the chromosome of *Acinetobacter radioresistens*, a commensal species of the human skin [32]. In this study, 11 out of 66 imipenem-resistant isolates harboured *bla*_OXA-23 _like gene. Attempts to transfer carbapenem resistance by electroporation of plasmid DNA from *bla*_OXA-23 _positive isolates were unsuccessful, indicating the probable chromosomal location of this gene.

Numerous reports on *A. baumannii *clinical isolates harbouring CHDLs OXA-58, OXA-40 and OXA-24 in the United States reflect their emergence as important carbapenemases [13,14,16]; however, they could not be detected in this set of isolates. Also, as demonstrated these *A. baumannii *clinical isolates (79.5%) possessed the chromosomal-encoded oxacillinase gene *bla*_OXA-66 _that encodes a β-lactamase known to confer carbapenemase properties [5,6]. Multiple copies of IS*Aba1 *are present in most isolates of *Acinetobacter spp*. [11]. It serves an important role as a 'mobile promoter' [33]. As evidence of this role, we found IS*Aba1 *immediately upstream of *bla*_OXA-66 _gene in 66 imipenem resistant isolates. More than 25 varieties of AmpC β-lactamases that share 94% protein sequence identity have been described for *Acinetobacter *spp. so far [34]. The oxyimino-β-lactam resistance seen in these *A. baumannii *strains is attributed to the presence of *bla*_ADC-25_. In this current study, gene cassettes including *aacA4*-*catB8-aadA1 *and *aacC1*-orfX-orfX'-*aadA1 *were predominantly found. Notably, so far these groups of cassettes are reported only in two international lineages, called European clones I and II [35-37]. Given the very high rate of quinolone resistance, this class is unlikely to have any clinical role in the treatment of MDR *A. baumannii *in central Ohio hospitals.

## Conclusion

Despite the increased frequency of multidrug resistance in *A. baumannii *in the United States, there exists a relative paucity of information regarding antimicrobial resistance in this Gram negative bacillus from central Ohio. The identification of *bla*_OXA-23 _and IS*Aba1 *associated *bla*_OXA-66 _genes in this study confirms the wide geographical distribution of carbapenemases among *A. baumannii *as well as their parallel appearance in outbreak strains. The experience with these MDR isolates suggested that surveillance for multidrug resistant *A. baumannii *should be maintained and careful infection control measures and cautious use of antibiotics must be promoted.

## Competing interests

The authors declare that they have no competing interests.

## Authors' contributions

VBS, GR, PP and DT performed the experiments, analyzed the data and drafted the paper. PP, KS, MM provided the strains, related clinical informations and susceptibility data. WG designed the idea, strategies to execute them, finalized the manuscript and provided intellectual suggestions. We thank all the anonymous reviewers for their fruitful suggestions and helpful comments, which helped us to significantly improve the manuscript.

## Supplementary Material

Additional file 1 **Phenotypic and genotypic characteristics of multidrug resistant clinical isolates of *A. baumannii***. ^a ^MIC for antibiotics are expressed in μg/ml. Abbreviations used for different drugs are: IPM: Imipenem, CAZ: Ceftazidime, KAN: Kanamycin, STR: Streptomycin, GEN: Gentamicin, AMK: Amikacin, CIP: Ciprofloxacin. Interpretation of the results was done using the criteria recommended by the Clinical and Laboratory Standards Institute CLSI [20]. *Escherichia coli *ATCC 25922 was used for quality control. ^b ^β-lactamases SHV, CTX-M, PER, SIM, IMP, VIM, GIM, OXA-24 like and OXA-58 like were not identified in this study. ^c ^The different gene cassettes (Type I to IV; as described in Figure 2) identified in the variable region of class 1 integron. ^d ^aminoglycoside phosphotransferase gene. ^e ^Reduction in MIC after the treatment of CCCP is given in parentheses. ^f ^MIC for NAL in strains with both mutations were >128 μg/ml. ^g ^Effect of inhibitors reserpine, verapamil (R/V) and CCCP on drug accumulation. +ve sign indicates reduction in MIC of ciprofloxacin on adding CCCP (25 μg/ml) while -ve sign indicates no change in MIC after the addition of either reserpine or verapamil in independent experiments. Efflux pump inhibitors used in this study had no intrinsic antibacterial activity against clinical isolates at the concentration used in the MIC determining experiments. Plasmid borne quinolone resistance *qnr *genes (*qnrA*_1–6_, *qnrB*_1–6 _and *qnrS*_1–2_) were not found in this study. ^h ^Strains that harbored *tet*(B) had an MIC >30 μg/ml towards tetracycline, none had *tet*(A). Click here for file
