# Supplementary material for: Genetic relatedness and molecular characterization of multidrug resistant Acinetobacter baumannii isolated in central Ohio, USA
Source: Ann Clin Microbiol Antimicrob. 2009 Jun 17;8:21. doi: 10.1186/1476-0711-8-21 (PMC2705342; doi:10.1186/1476-0711-8-21)
Supplement: Additional file 1 — Phenotypic and genotypic characteristics of multidrug resistant clinical isolates of A. baumannii. a MIC for antibiotics are expressed in μg/ml. Abbreviations used for different drugs are: IPM: Imipenem, CAZ: Ceftazidime, KAN: Kanamycin, STR: Streptomycin, GEN: Gentamicin, AMK: Amikacin, CIP: Ciprofloxacin. Interpretation of the results was done using the criteria recommended by the Clinical and Laboratory Standards Institute CLSI [20]. Escherichia coli ATCC 25922 was used for quality control. b β-lactamases SHV, CTX-M, PER, SIM, IMP, VIM, GIM, OXA-24 like and OXA-58 like were not identified in this study. c The different gene cassettes (Type I to IV; as described in Figure 2) identified in the variable region of class 1 integron. d aminoglycoside phosphotransferase gene. e Reduction in MIC after the treatment of CCCP is given in parentheses. f MIC for NAL in strains with both mutations were >128 μg/ml. g Effect of inhibitors reserpine, verapamil (R/V) and CCCP on drug accumulation. +ve sign indicates reduction in MIC of ciprofloxacin on adding CCCP (25 μg/ml) while -ve sign indicates no change in MIC after the addition of either reserpine or verapamil in independent experiments. Efflux pump inhibitors used in this study had no intrinsic antibacterial activity against clinical isolates at the concentration used in the MIC determining experiments. Plasmid borne quinolone resistance qnr genes (qnrA1–6, qnrB1–6 and qnrS1–2) were not found in this study. h Strains that harbored tet(B) had an MIC >30 μg/ml towards tetracycline, none had tet(A). [file 1476-0711-8-21-S1.doc]

| **No.** | **PFGE** | **β lactams** | | | | | | | **Aminoglycosides** | | | | | | | | **Quinolones^f^** | | | | | |  |
| --- | --- | --- | --- | --- | --- | --- | --- | --- | --- | --- | --- | --- | --- | --- | --- | --- | --- | --- | --- | --- | --- | --- | --- |
|  |  | **MIC**^a^ | | **β-lactamases**^b^ | | | | | **MIC**^a^ | | | | **Integrons** | | | ***aphA6*^d^** | **MIC**^a^ | **Mutations** | | **Efflux^g^** | | **Efflux genes** | |
|  |  | **IPM** | **CAZ** | ***bla*_TEM-1_** | ***bla*_OXA-23_** | ***bla_ADC-25_*** | ***bla*_OXA-66_** | **IS*_Aba1_*** | **KAN** | **STR** | **GEN** | **AMK** | ***int*I** | **Size** | **Variable^c^** |  | **CIP^e^** | ***gyrA*** | ***parC*** | **R/V** | **CCCP** | ***adeB*** | ***tetB*^h^** |
|  |  |  |  |  |  |  |  |  |  |  |  |  |  |  |  |  |  |  |  |  |  |  |  |
| 1 | I | 10 | 0.2 | - | - | - | + | + | >8 | >16 | >8 | >128 | + | 1.6 | Type III | - | >72 (>10) | S83L | S80L | - | + | + | + |
| 1 | I | 6 | 0.2 | - | - | - | + | + | >8 | >16 | >8 | >128 | + | 1.6 | Type III | - | >52 (<1) | S83L | S80L | - | + | - | + |
| 1 | I | 10 | 32 | - | - | + | + | + | <4 | >16 | >8 | >128 | + | 1.6 | Type III | - | >72 (>10) | S83L | S80L | - | + | + | + |
| 1 | I | 4 | 32 | + | - | + | + | + | <2 | >8 | 2 | >128 | - | - | - | - | >32 (<1) | S83L | S80L | - | + | - | + |
| 1 | II | 4 | 32 | - | - | + | + | + | <2 | >2 | <1 | >128 | - | - | - | - | >52 (<1) | S83L | S80L | - | + | - | + |
| 2 | III | 6 | 32 | - | - | + | + | + | <2 | >2 | 4 | >256 | - | - | - | + | >72 (>10) | S83L | S80L | - | + | + | + |
| 2 | IV | 4 | 32 | + | - | + | + | + | >8 | >16 | >8 | >128 | + | 2.5 | Type I | - | >72 (>10) | S83L | S80L | - | + | + | + |
| 1 | V | 10 | 32 | + | + | + | + | + | <4 | >16 | >8 | >256 | + | 2.3 | Type II | - | >32 (<1) | S83L | S80L | - | + | - | + |
| 1 | V | 10 | 32 | + | - | + | + | + | >8 | >16 | >8 | >256 | + | 2.3 | Type II | - | >72 (>10) | S83L | S80L | - | + | + | + |
| 1 | VI | 4 | 32 | + | - | + | + | + | <2 | >16 | >8 | >256 | + | 2.3 | Type II | - | >52 (<1) | S83L | S80L | - | + | - | + |
| 19 | VII | 10 | 32 | + | - | + | + | + | >8 | >16 | >8 | >256 | + | 2.3 | Type II | - | >72 (>10) | S83L | S80L | - | + | + | + |
| 1 | VII | 10 | 32 | - | - | + | + | + | <2 | >16 | >8 | >256 | + | 2.3 | Type II | - | >72 (>10) | S83L | S80L | - | + | + | + |
| 7 | VII | 4 | 32 | - | - | + | + | + | <2 | >8 | 2 | >256 | - | - | - | - | >72 (>10) | S83L | S80L | - | + | + | + |
| 1 | VII | >16 | 32 | - | + | + | + | + | <4 | >2 | <1 | >256 | - | - | - | + | >72 (>10) | S83L | S80L | - | + | + | + |
| 1 | VIII | 4 | 32 | + | - | + | + | + | <2 | >16 | >8 | >256 | + | 2.5 | Type I | + | >32 (<1) | S83L | S80L | - | + | - | + |
| 1 | IX | 4 | 32 | + | - | + | + | + | <2 | >2 | <1 | >128 | - | - | - | - | >32 (<1) | S83L | S80L | - | + | - | + |
| 1 | X | 6 | 32 | - | - | + | + | + | >8 | >8 | 4 | >128 | - | - | - | - | >52 (<1) | S83L | S80L | - | + | - | + |
| 3 | XI | 4 | 32 | - | - | + | + | + | <2 | >16 | >8 | >256 | + | 2.3 | Type II | - | >72 (>10) | S83L | S80L | - | + | - | + |
| 1 | XII | 10 | 32 | - | + | + | + | + | <2 | >2 | <1 | >256 | - | - | - | + | >32 (<1) | S83L | S80L | - | + | - | + |
| 1 | XIII | 4 | 0.2 | - | - | - | + | + | <4 | >16 | >8 | >256 | + | 0.8 | Type IV | + | >72 (>10) | S83L | S80L | - | + | - | + |
| 7 | ND | >16 | 32 | - | + | + | + | + | >8 | >8 | <1 | >256 | - | - | - | + | >72 (>10) | S83L | S80L | - | + | + | + |
| 3 | ND | 4 | 32 | + | - | + | + | + | <2 | >2 | 2 | >128 | - | - | - | - | >32 (<1) | S83L | S80L | - | + | - | + |
| 1 | ND | 6 | 0.2 | - | - | - | + | + | <4 | >2 | 4 | >128 | - | - | - | - | >52 (<1) | S83L | S80L | - | + | - | + |
| 1 | ND | 4 | 32 | - | - | + | + | + | <2 | >2 | <1 | >128 | - | - | - | - | >32 (<1) | S83L | S80L | - | + | - | + |
| 1 | ND | 10 | 32 | - | - | + | + | + | >8 | >16 | >8 | >256 | - | - | - | + | >72 (>10) | S83L | S80L | - | + | + | + |
| 1 | ND | 4 | 32 | - | - | + | + | + | <2 | >8 | 4 | >256 | - | - | - | + | >32 (<1) | S83L | S80L | - | + | - | + |
| 1 | ND | 4 | 32 | - | - | + | + | + | <4 | >2 | 2 | >128 | - | - | - | - | >52 (<1) | S83L | S80L | - | + | - | + |
| 1 | ND | 4 | 32 | - | - | + | + | + | <2 | >8 | 2 | >128 | - | - | - | - | >32 (<1) | S83L | S80L | - | + | - | + |
| 1 | ND | >16 | 32 | - | + | + | + | + | <4 | >16 | >8 | >128 | - | - | - | - | >72 (>10) | S83L | S80L | - | + | + | + |
| 1 | ND | 4 | 32 | - | - | + | + | + | <2 | >2 | <1 | >128 | - | - | - | - | >72 (>10) | S83L | S80L | - | + | - | + |
